# 

**Published:** 1897-03

**Authors:** 


					﻿Vol. XVII.
MARCH, 1897.
NO. 3?
THE
pDMCEOPATHlC pHY£lClA]\l,
A Monthly Journal of Homoeopathic Materia Medica
and Clinical Medicine.
“ He it a freeman whom truth makes free, and all are slaves beside."
"Seek the Truth: come whence it may, cost what it will."
EDITED AND PUBLISHED BY
WALTER M. JAMES, M. D.
PHILADELPHIA:
ZCTo. 1231 LOCUST STREET.
ii.pjKriSb n f
ALFRED HEATH & CO., Sole Agents foe Great Britain,
11A TZVmywtt	Cmiora
Yearly Subscription, $2.50, invariably in advance. Single numbers^ 25 cts.
Entered *t th* Philadelphia. Pn«t-(lflfic* aa R*ftnnd-Cl aia Mail Matter.
				

